# Enhancing Water and Soil Resources Utilization via Wolfberry–Alfalfa Intercropping

**DOI:** 10.3390/plants13172374

**Published:** 2024-08-26

**Authors:** Jinghai Wang, Minhua Yin, Yaya Duan, Yanbiao Wang, Yanlin Ma, Heng Wan, Yanxia Kang, Guangping Qi, Qiong Jia

**Affiliations:** 1 College of Water Conservancy and Hydropower Engineering, Gansu Agricultural University, Lanzhou 730070, China; wangjh@gsau.edu.cn (J.W.); yinmh@gsau.edu.cn (M.Y.); 18292757072@163.com (Y.D.); 17794437957@163.com (Y.W.); yanxiakang@gsau.edu.cn (Y.K.); qigp@gsau.edu.cn (G.Q.); jiaq@gsau.edu.cn (Q.J.); 2 Ministry of Education/College of Water Resources and Architectural Engineering, Northwest A&F University, Yangling 712100, China

**Keywords:** model simulation, crop coefficients, soil evaporation, transpiration, water-saving mechanism, wolfberry (*Lycium barbarum* L.), Alfalfa (*Medicago sativa* L.)

## Abstract

The impact of the intercropping system on the soil–plant–atmosphere continuum (SPAC), encompassing soil evaporation, soil moisture dynamics, and crop transpiration, remains an area of uncertainty. Field experiments were conducted for two years in conjunction with the SIMDualKc (Simulation Dual Crop Coefficient) model to simulate two planting configurations: sole-cropped wolfberry (*Lycium barbarum* L.) (D) and wolfberry intercropped with alfalfa (*Medicago sativa* L.) (J). These configurations were subjected to different irrigation levels: full irrigation (W1, 75–85% θfc), mild deficit irrigation (W2, 65–75% θfc), moderate deficit irrigation (W3, 55–65% θfc), and severe deficit irrigation (W4, 45–55% θfc). The findings revealed that the JW1 treatment reduced the annual average soil evaporation by 32% compared with that of DW1. Additionally, mild, moderate, and severe deficit irrigation reduced soil evaporation by 17, 24, and 36%, respectively, compared with full irrigation. The intercropping system exhibited a more efficient canopy structure, resulting in reduced soil evaporation and alleviation of water stress to a certain extent. In terms of temporal dynamics, monocropping resulted in soil moisture levels from 1% to 15% higher than intercropping, with the most significant differences manifesting in the mid to late stages, whereas differences in the early stages were not statistically significant. Spatially, the intercropping system exhibited 7–19% lower soil water contents (SWCs) than sole cropping, primarily within the root water uptake zone within the 0–60 cm soil layer. The intercropping system showed an enhanced water absorption capacity for plant transpiration, resulting in a 29% increase in transpiration compared with sole cropping, thereby achieving water-saving benefits. These findings contribute to our understanding of the agronomic and environmental implications of intercropping wolfberry and alfalfa in arid regions and provide insights into optimizing water and soil resource management for sustainable agricultural practices.

## 1. Introduction

Wolfberry (*Lycium barbarum* L.) is a perennial deciduous shrub belonging to the Solanaceae family. It has a rich cultivation history spanning over a millennium in China. Renowned for its robust traits, such as salt and alkali resistance, adaptability to infertile soils, drought tolerance, and high economic returns [1], wolfberry is a significant agricultural commodity. The arid and semi-arid regions of Northwest China have emerged as primary production hubs for this remarkable crop owing to favorable climatic conditions characterized by ample sunlight and notable day–night temperature fluctuations [2]. As the market demand for wolfberry continues to surge and local efforts to harness saline–alkali lands intensify, the cultivation footprint of wolfberry has consistently expanded. However, the current state of wolfberry cultivation in China remains in a developmental phase, marked by monocropping, meager vegetation cover, severe soil evaporation, and extensive field management practices. In tandem, these factors exacerbate secondary soil salinization. Intercropping systems featuring the harmonious coexistence of woody plants and grasses have emerged as a flagship approach to eco-agriculture [3]. They stand out as a means to effectively curtail soil evaporation, elevate water utilization efficiency, and bolster economic return [4]. Alfalfa (*Medicago sativa* L.), celebrated as the “forage king” and “feed queen”, emerged as a natural choice for intercropping with wolfberry. Its robust regenerative abilities, palatability, and rich reservoir of high-quality crude protein render it an essential part of wolfberry-based agroforestry systems [5]. Thus, investigation of the water-saving mechanisms within the wolfberry–alfalfa intercropping system is pivotal for the sustainable evolution of agroforestry practices.

Agroforestry systems that combine woody and herbaceous plants are widely implemented across various regions, including Europe, South America, the Saharan region of Africa, and South Asia. These systems exhibit diverse forms owing to variations in climatic conditions and management practices [6,7,8]. Within agroforestry systems, the interplanting of herbaceous vegetation between woody plants in alleys optimizes land and solar resource utilization. This multifaceted approach not only enhances land coverage but also mitigates soil evaporation, fostering improved vegetation structure and soil enrichment strategies in line with the adage, “grasses to ameliorate the soil, and grasses to stimulate the trees” [8,9,10]. The interaction between these two components in agroforestry systems introduces variations in soil moisture, soil evaporation, and crop transpiration. These changes are influenced by factors such as the choice of intercropped vegetation, prevailing climatic conditions, soil texture, and field management practices. Several studies suggest that, although herbaceous plants in agroforestry systems consume some soil moisture, they also contribute significantly to increased rainfall infiltration, reduced soil evaporation, and minimized soil erosion, ultimately resulting in stable or even augmented soil moisture levels [11,12]. For instance, in the case of guinea grass (*Panicum maximum* Jacq.) intercropped with four tree species at varying row spacings, medium row spacing consistently demonstrated the highest soil moisture levels, accompanied by optimal growth and yields [13]. Wen et al. [14] observed that intercropping of *Alpinia oxyphylla* Miq. (a medicinal herb) in oak plantations exhibited variable effects on water consumption rates and uptake across different soil layers, ultimately achieving an equilibrium in soil moisture distribution and overall soil moisture enhancement. In a semi-arid region of Liaoning, China, Bai et al. [15] reported that apricot (*Prunus armeniaca* L.) orchard intercropping systems positively influenced soil moisture conditions compared with sole cropping. Nonetheless, Oswald et al. [16] found that intercropping grass-legume mixtures with young peach (*Prunus persica* (L.) Batsch) trees in southern France led to more severe water stress than sole cropping, particularly during early growth when soil cover fabric was used. However, judicious intercrop cutting effectively mitigates water stress during mid- to late-growth stages [17]. Similarly, Kurt et al. [18] documented that in a switchgrass (*Panicum virgatum* L.) and juvenile loblolly pine (*Pinus taeda* L.) intercropping system, competition exerted a greater negative impact than positive effects, resulting in reduced soil moisture and decreased yields for both switchgrass and juvenile loblolly pine when compared with sole cropping. In jujube (*Ziziphus jujuba* Mill.) orchards on the Chinese Loess Plateau, intercrop species and field management practices significantly influence the root distribution of agroforestry systems, thereby affecting the soil moisture conditions [19]. These findings underscore the need to consider various factors when evaluating the impact of agroforestry systems on soil moisture dynamics, emphasizing the necessity of context-specific approaches to optimize water resource management within agroforestry practices.

Soil moisture plays a pivotal role in agroecosystems and is primarily influenced by two main consumption pathways: soil evaporation and crop transpiration. The introduction of intercropping into these systems reshapes the spatial distribution of plant canopies, thereby exerting a profound influence on the partitioning of soil evaporation and crop transpiration [20]. Notably, intercropping often features taller crops, which, owing to their increased stature, can absorb more solar radiation, consequently leading to higher rates of transpiration when compared with shorter companion crops. For instance, a study conducted in the Hetao Irrigation District of Inner Mongolia revealed that maize (*Zea mays* L.), a tall crop, exhibited a substantial increase in transpiration, ranging from 29.3% to 42.1%, compared with shorter tomato plants [19]. This difference in transpiration is predominantly attributed to belowground resource competition, with the root system of maize demonstrating a water uptake capacity 7.3 times greater than that of tomatoes (*Solanum lycopersicum* L.) [21]. To better understand the nuances of soil moisture dynamics, Wang et al. [22] used an enhanced modeling approach to simulate transpiration allocation within agroforestry systems. Their findings indicated that apple trees (*Malus pumila* Mill.), with their greater stature, consumed 33.3–48.0% more water than cocksfoot grass (*Dactylis glomerata* L.). However, it is crucial to emphasize that the effect of intercropping on soil moisture is not universally consistent. For instance, in wheat (*Triticum aestivum* L.) and maize intercropping, adequate underground interaction between crop roots is conducive to reducing water consumption. Increasing maize planting density is beneficial to reduce the water consumption of the long-term subsurface complete interaction between wheat and maize [23]. Within the agroforestry systems (crop + shrub) situated in central and southern Senegal, the introduction of shading mechanisms reduces direct solar exposure to the soil surface. After rainfall, there is an initial increase in the soil moisture content, followed by a period of pronounced soil evaporation, with a subsequent gradual decline in the rate of evaporation [24]. In semi-arid tropical grasslands where taller trees predominate, the higher proportion of above-ground woody structures reduces evaporation due to “savings” from reduced solar exposure, significantly surpassing the “losses” from tree transpiration. In contrast, agroforestry systems, such as orchards, which are characterized by densely planted, shorter trees, experience a counterbalancing effect due to canopy interception and increased transpiration. Consequently, these dynamics can offset the favorable microclimatic effects. In pursuit of maximum productivity and economic returns, farmers tend to favor economically profitable fruit trees and similar choices [25]. It is important to emphasize that soil surface water evaporation is a pivotal component of soil moisture balance, particularly in areas with sparse vegetation, where young trees may struggle to provide complete ground cover. This results in substantial soil moisture loss through ineffective evaporation mechanisms [26]. Although intercropping has the potential to reduce soil evaporation within tree rows, it may also intensify soil transpiration [27]. Radersma and Ong [28] conducted research in Kenya and reported a 35% reduction in soil evaporation within agroforestry systems compared with bare soil. However, Wang et al. [29] found that intercropping forage grass with jujube orchards in the hilly Loess Plateau partially offset reduced soil evaporation and increased infiltration, resulting in a certain degree of negative impact on soil moisture conditions. Effective field management practices such as timely grass-cutting have been identified as viable solutions for mitigating this impact. In conclusion, the perspectives of researchers, both nationally and internationally, regarding the influence of intercropping systems on soil water content (SWC), soil evaporation, and crop transpiration remain diverse. These disparities emphasize the necessity of region-specific analyses that account for factors such as climate conditions, crop types, and field management practices. Importantly, the distinctive wolfberry–alfalfa intercropping system, particularly in the arid regions of Northwestern China, is not well-represented in the literature. Therefore, it is imperative to elucidate the spatial distribution of soil moisture within the wolfberry–alfalfa intercropping system, quantify soil evaporation and crop transpiration, explore their intricate interactions, and uncover the mechanisms governing soil-crop water regulation within the soil–plant–atmosphere continuum (SPAC). This undertaking will establish a robust theoretical foundation underpinning rational irrigation practices and sustainable advancement of the wolfberry–alfalfa intercropping system.

Therefore, the aims of this study were to (1) utilize the SIMDualKc (Simulation Dual Crop Coeffcient) model for simulation and validation to demonstrate its applicability in the study area and determine crop coefficients for each growth stage; (2) define the soil evaporation dynamics during the crop growth stages and quantitatively assess the influence of intercropping on soil evaporation; (3) compare the spatiotemporal variations in soil moisture between intercropping and sole cropping; and (4) determine the mechanisms governing soil–plant water dynamics and quantitatively assess the water-saving benefits of intercropping by evaluating crop transpiration across growth stages and their proportion to evaporation, in conjunction with the interactive impacts on soil evaporation and moisture. Through a comprehensive analysis of the water-saving mechanisms at play, we hope to provide valuable insights to the scientific community and promote the adoption of eco-friendly and economically viable agroforestry practices.

## 2. Results

### 2.1. Calibration and Validation of the SIMDualKc (Simulation Dual Crop Coefficient)

The model parameters initially adopted the recommended values from FAO-56 [30]. Adjustments were made to the parameters during the model calibration process, resulting in a close match between the SWCs (soil water contents) and observed values (Table 1). For alfalfa, a crop that can be harvested multiple times during the growing season, these parameters represent the crop coefficients for each subsequent growth period. K_cb_ ini signifies the crop coefficient after the previous harvest, whereas K_cb_ end represents the crop coefficient before the next harvest.

**Figure 1:**
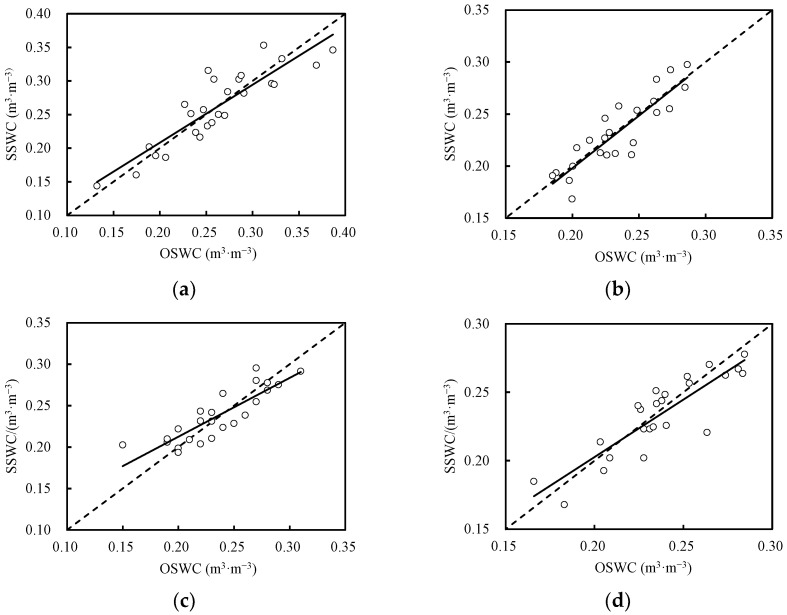
Comparison of the measured and simulated soil moisture by calibration and validation of the model for Wolfberry monoculture treatment (**a**,**c**) in 2019 and 2020, respectively, intercropping between wolfberry and alfalfa (**b**,**d**) in 2019 and 2020. SSWC, simulated soil water content; OSWC, observed soil water content.

Figure 1 illustrates the observed soil water contents (OSWCs) and simulated soil water contents (SSWCs) for both wolfberry monoculture and wolfberry–alfalfa intercropping under full irrigation treatments. The simulated values for all the treatments were evenly distributed around the 1:1 line. In 2019, for both intercropping and monoculture treatments, “b” values were approximately 0.99, with R-squared (R^2^) values ranging from 0.88 to 0.89. The root mean square error (RMSE) varied from 0.0163 to 0.0267 m^3^ m^−3^, whereas the index of agreement (d_IA_) ranged from 0.92 to 0.93. The Nash-Sutcliffe Efficiency (EF) remained consistently at 0.70. These results indicated a high level of calibration accuracy. When validated with 2020 data, “b” values ranged from 0.98 to 1.00, and R^2^ values ranged from 0.86 to 0.87. The RMSE fell within the range of 0.0151–0.0193 m^3^ m^−3^, whereas the d_IA_ values ranged from 0.92 to 0.93. In summary, the soil moisture simulations generated by the SIMDualKc model met the required criteria for accuracy, as detailed in the Table 2 of model validation parameters.

### 2.2. Comparison of Crop Coefficients

**Figure 2:**
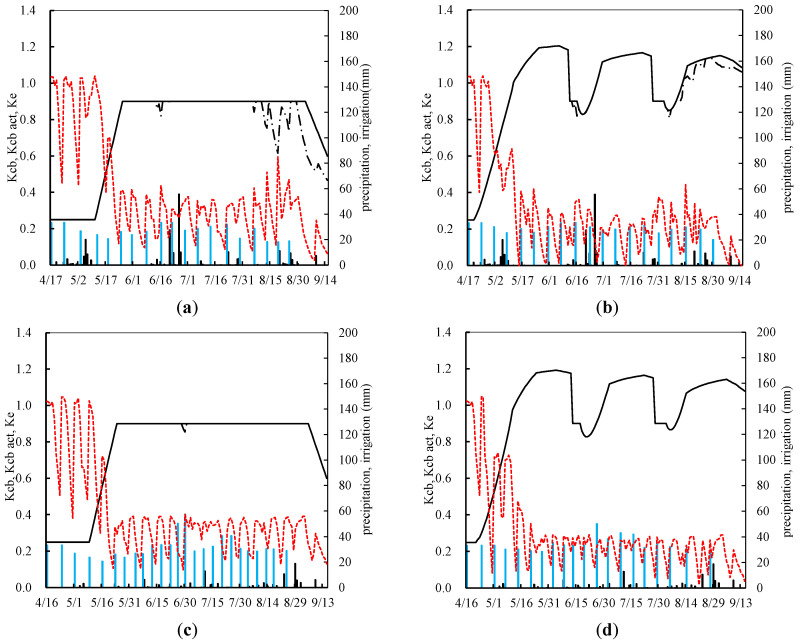
Standard and actual basal crop coefficient (K_cb_, K_cb act_), evaporation coefficient (K_e_) for wolfberry monoculture in 2019 (**a**), 2020 (**c**) and intercropping between wolfberry and alfalfa in 2019 (**b**), 2020 (**d**), with depicting precipitation and irrigation events. The black polyline represents k_cb_, the black dashed line represents K_cb act_, the red dashed line represents K_e_, the black vertical line represents precipitation, and the blue vertical line represents irrigation.

In the wolfberry monoculture treatment (Figure 2a,c), plant growth was sluggish during the initial growth stage, characterized by relatively small leaves and weak transpiration, resulting in a base crop coefficient (K_cb ini_) of 0.25. As the growth progressed to the rapid growth phase, leaf development accelerated, transpiration intensified, and the crop coefficient exhibited linear growth. By the mid-growth stage, the base crop coefficient (K_cb mid_) had reached 0.9. During the early- to mid-growth stage, when the root system remains relatively shallow, soil moisture satisfactorily meets the plant’s growth requirements, which leads to a close alignment between K_cb_ and K_cb act_. As the late growth stage is set in, plant growth decelerates, and leaves turn yellow and drop, resulting in a reduction in the crop coefficient, with a base crop coefficient (K_cb end_) of 0.6. In the late growth stage, after both plant and fruit development are complete, water consumption decreases and corresponding reductions in irrigation are made to prevent water wastage. In 2019 (Figure 2a), the late growth stage witnessed lower rainfall, causing water stress. Nevertheless, it has been concluded that plant and fruit development minimally affect the growth and yield of wolfberry. The soil evaporation coefficient (K_e_) varied in response to irrigation and rainfall. During the early growth stage, when leaves are not fully developed and ground cover is limited, water consumption is predominantly driven by soil evaporation, resulting in relatively high K_e_ values. Following irrigation and rainfall events, K_e_ rapidly increased owing to strong evaporation, leading to elevated surface soil moisture content. Subsequently, soil evaporation entered an energy-limited phase, marked by evaporation occurring at its maximum rate, which induced significant K_e_ fluctuations. In the mid-growth stage, as the leaves become nearly fully developed and ground cover increases, transpiration emerges as the primary water consumption process, causing K_e_ to decrease and exhibit reduced fluctuations. The study findings indicated that, for the wolfberry monoculture, K_e_ ranged from 0.43 to 1.02 during the initial growth stage and subsequently decreased to a range of from 0.12 to 0.41 from the mid-growth stage onward.

For the wolfberry and alfalfa intercropping systems (Figure 2b,d), the dynamic interplay and competition between the two components introduced complexity to the variation in crop coefficients. Owing to the growth characteristics of alfalfa, which shorten the initial growth period of the intercropping system, it undergoes three harvests during its entire growth cycle. Consequently, the crop coefficient for the intercropping system exhibited a periodic three-phase variation curve, with each harvest representing the growth cycle. The overall trend of the crop coefficient within each growth cycle was similar to that of monoculture. During the initial growth stage of the first alfalfa harvest in the intercropping system, the base crop coefficient (K_cb ini_) was relatively low, averaging 0.25. In the rapid growth phase, the crop coefficient increased linearly, reaching its maximum value, averaging 1.19, when the ground was completely covered by vegetation. After the alfalfa harvest, the base crop coefficient decreased sharply to 0.9. As alfalfa began to regrow, there was a brief decrease in the intercropping crop coefficient. This temporary decrease was due to the initial low height of the alfalfa plants, resulting in a significant difference in height between alfalfa and wolfberry, which negatively affected the intercropping crop coefficient. With the rapid growth of alfalfa, it entered the rapid growth phase. When the ground was completely covered by the second alfalfa harvest, the base crop coefficient reached a maximum value of 1.15. These results indicated that the time spans of the second and third harvest phases were longer than that of the first harvest phase. After the second harvest (early stage of the third harvest), K_cb_ was 0.9; when the ground was fully covered during the third harvest, it reached 1.13. Before harvest (late growth stage), K_cb_ was 1.08. In 2019 (b), the intercropping treatment also experienced mild water stress during the late growth stage, although the stress level was significantly lower than that of the wolfberry monoculture treatment. This reduction in stress can be attributed to intercropping, which increases ground cover and reduces soil evaporation, helping maintain adequate moisture levels in the root zone. Therefore, intercropping could alleviate a certain degree of water stress. The soil evaporation coefficient (K_e_) exhibited a similar trend to that of the monoculture treatment during the growth period. However, owing to the higher ground cover during intercropping, soil evaporation was reduced, resulting in smaller fluctuations. For wolfberry–alfalfa intercropping, K_e_ ranged from 0.41 to 1.02 during the initial growth stage and decreased to a range of from 0.06 to 0.21 from the mid-growth stage onward. The interannual differences in the crop coefficients between 2019 and 2020 were relatively small.

### 2.3. Comparison of Soil Evaporation

**Figure 3:**
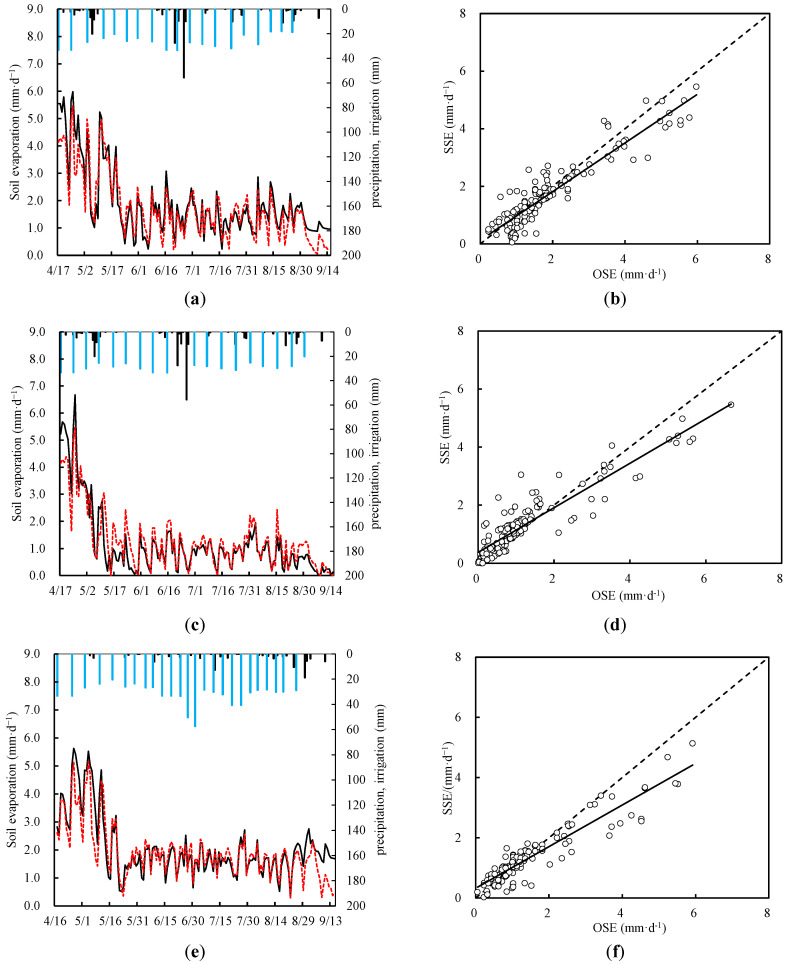

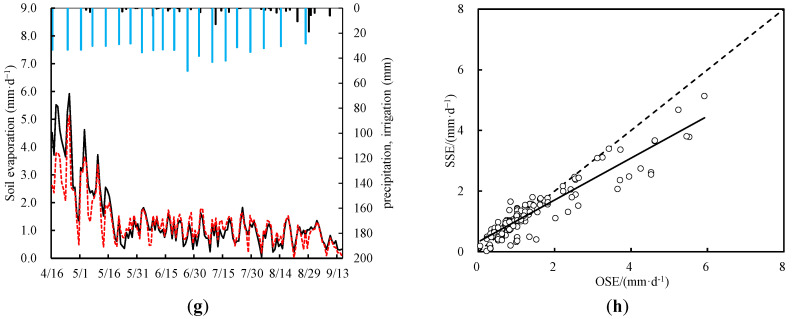
Comparison between simulated and observed soil evaporation: on the left, the changing progress of the observed and simulated soil evaporation for wolfberry monoculture (**a**,**e**) and intercropping between wolfberry and alfalfa (**c**,**g**) in 2019 and 2020; The figure on the right shows the regression between the observed and calculated values of soil evaporation from wolfberry monoculture (**b**,**f**) and intercropping between wolfberry and alfalfa (**d**,**h**) in 2019 and 2020. The red dotted line is the simulation of soil evaporation (SSE), and the black solid line is the observation of soil evaporation (OSE). The black vertical line represents precipitation, and the blue vertical line represents irrigation.

The soil evaporation simulations for both wolfberry monoculture and intercropping under full irrigation showed a consistent distribution around the 1:1 line (Figure 3). In 2019, for both intercropping and monoculture treatments, the Nash–Sutcliffe efficiency coefficient (b) ranged from 0.89 to 0.93, whereas the coefficient of determination (R^2^) ranged from 0.91 to 0.93. The RMSE varied from 0.51 to 0.54 m^3^ m^−3^, and the dIA ranged from 0.95 to 0.96. The EF was in the range of from 0.82 to 0.84. These results highlighted the high calibration accuracy that was achieved. Validation using data from 2020 yielded similar outcomes, with b ranging from 0.83 to 0.89 and R^2^ ranging from 0.87 to 0.93. The RMSE varied from 0.50 to 0.55 m^3^ m^−3^, and the dIA ranged from 0.92 to 0.94 (Table 3). The soil evaporation simulations based on the SIMDualKc model consistently met the required standards.

### 2.4. Soil Evaporation

**Figure 4:**
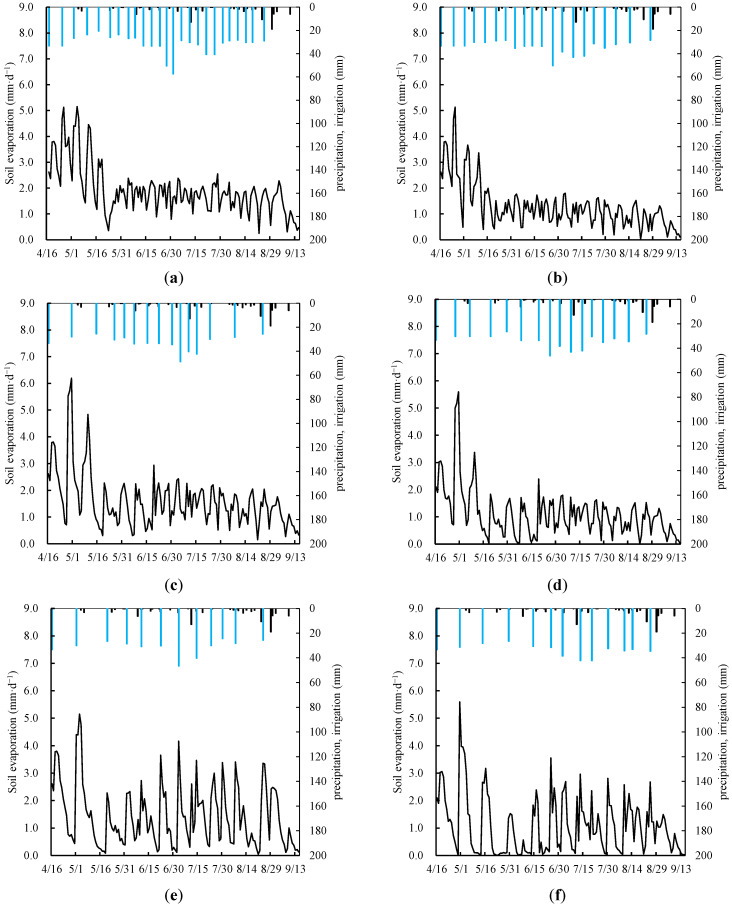

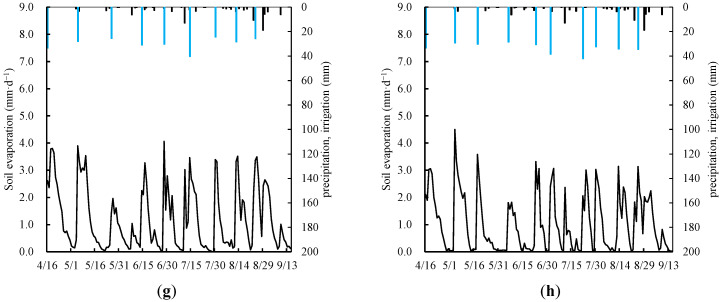
Simulated soil evaporation under different treatments: full irrigation (**a**), mild deficit (**c**), moderate deficit (**e**), and severe deficit (**g**), with wolfberry monoculture and full irrigation (**b**), mild deficit (**d**), moderate deficit (**f**), and severe deficit (**h**), with intercropping of wolfberry and alfalfa. The black vertical line represents precipitation, and the blue vertical line represents irrigation.

In both 2019 and 2020, whether in the wolfberry monoculture or intercropped with alfalfa, the soil evaporation trends exhibited consistent patterns. Using 2020 as an example (Figure 4), all treatment groups demonstrated similar behavior in soil evaporation, characterized by higher values during the early growth stage and lower values in the later stages of crop development. Throughout the growing season, soil evaporation followed a pulsating curve, with fluctuations corresponding to rainfall and irrigation events. Among the different irrigation treatments, the curve for the fully irrigated conditions exhibited the smallest fluctuations. As the severity of the water deficit increased, soil evaporation exhibited greater variability. This phenomenon is attributed to increased water stress, which adversely affects the growth and development of crops. Severe water stress inhibits leaf growth, resulting in reduced soil cover, and, consequently, increased soil evaporation. In particular, after irrigation or rainfall events, when shallow soil moisture levels were high, soil evaporation rapidly increased but then gradually decreased as soil moisture levels declined, contributing to more pronounced fluctuations in soil evaporation. Intercropping treatments displayed a more rational spatial distribution of components with higher soil coverage, effectively reducing soil evaporation and mitigating fluctuations during the growing season. In the DW1 treatment (wolfberry monoculture), the average soil evaporation was 292.48 mm, whereas, in the JW1 treatment (wolfberry intercropped with alfalfa), it was 198.68 mm. Intercropping reduced ineffective soil evaporation by 32% compared with monoculture. In the monoculture treatments, soil evaporation decreased with increasing water deficits. Relative to fully irrigated conditions, soil evaporation decreased by 17% under mild deficit irrigation, 24% under moderate deficit irrigation, and 36% under severe deficit irrigation. However, there were no significant differences in soil evaporation among the intercropping treatments under different water deficit levels, indicating that intercropping can alleviate water stress through the interaction of different components.

### 2.5. Impact of Intercropping on Spatiotemporal Soil Moisture Dynamics

**Figure 5:**
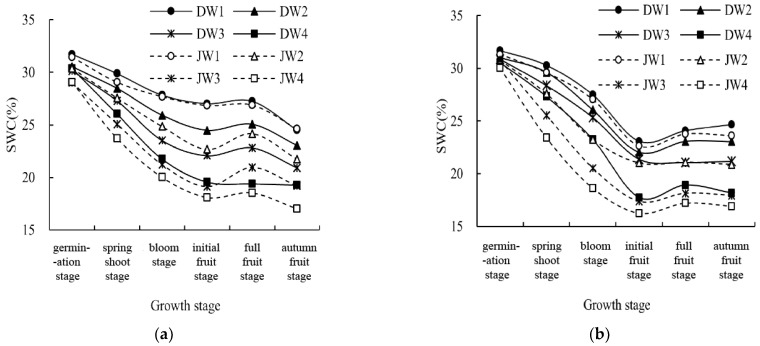
SWC variation during the growth period for different treatments in 2019 (**a**) and 2020 (**b**). DW1: wolfberry monoculture and full irrigation (75–85%θfc), DW2: wolfberry monoculture and mild deficit irrigation (65–75%θfc), DW3: wolfberry monoculture and moderate deficit irrigation (55–65%θfc), DW4: wolfberry monoculture and severe deficit irrigation (45–55%θfc); JW1: wolfberry intercropped with alfalfa and full irrigation (75–85%θfc), JW2: wolfberry intercropped with alfalfa and mild deficit irrigation (65–75%θfc), JW3: wolfberry intercropped with alfalfa and moderate deficit irrigation (55–65%θfc), JW4: wolfberry intercropped with alfalfa and severe deficit irrigation (45–55%θfc).

During the 2019–2020 growing season, a consistent pattern of soil moisture variation was observed as the cultivation of wolfberry progressed (Figure 5). SWCs initially decreased, followed by a gradual increase. In the early stages, particularly during germination, the SWCs were relatively high owing to ample spring irrigation, which ensured optimal wolfberry growth. However, as wolfberry plants matured, soil moisture levels declined, reaching their lowest levels during the initial fruiting stage. This decline corresponds to a crucial phase of fruit development marked by significant root water uptake, which facilitates fruit maturation. Among the DW treatments (DW1, DW2, DW3, and DW4), the average SWCs during the growth period ranged from 17.74% to 31.74%. For the JW treatments (JW1, JW2, JW3, and JW4), the average SWCs during the growth period varied from 16.25% to 31.44%. Increasing irrigation led to higher soil moisture levels, with DW1, DW2, and DW3 showing 23%, 16%, and 9% higher SWCs than DW4, respectively. Similarly, JW1, JW2, and JW3 exhibited 33%, 20%, and 7% higher SWCs than JW4 under increased irrigation. In general, single cropping (monoculture) resulted in higher soil moisture levels than intercropping. However, under sufficient irrigation conditions, no significant differences were observed between intercropping and single-cropping treatments. This lack of distinction can be attributed to the enhanced water absorption capacity of the intercropped plants through their root systems, rendering the advantage of intercropping less pronounced with ample irrigation. During the initial stages, when spring irrigation was sufficient, both single cropping and intercropping treatments exhibited relatively high soil moisture levels, with no significant differences. At this point, both wolfberry and intercropped alfalfa were in their early growth stages and characterized by relatively low water requirements. Nevertheless, as the growth period progressed, disparities between the single cropping and intercropping treatments gradually became more evident. Research findings indicate that soil moisture levels in single cropping treatments were consistently 1–15% higher than those in intercropping treatments.

**Figure 6:**
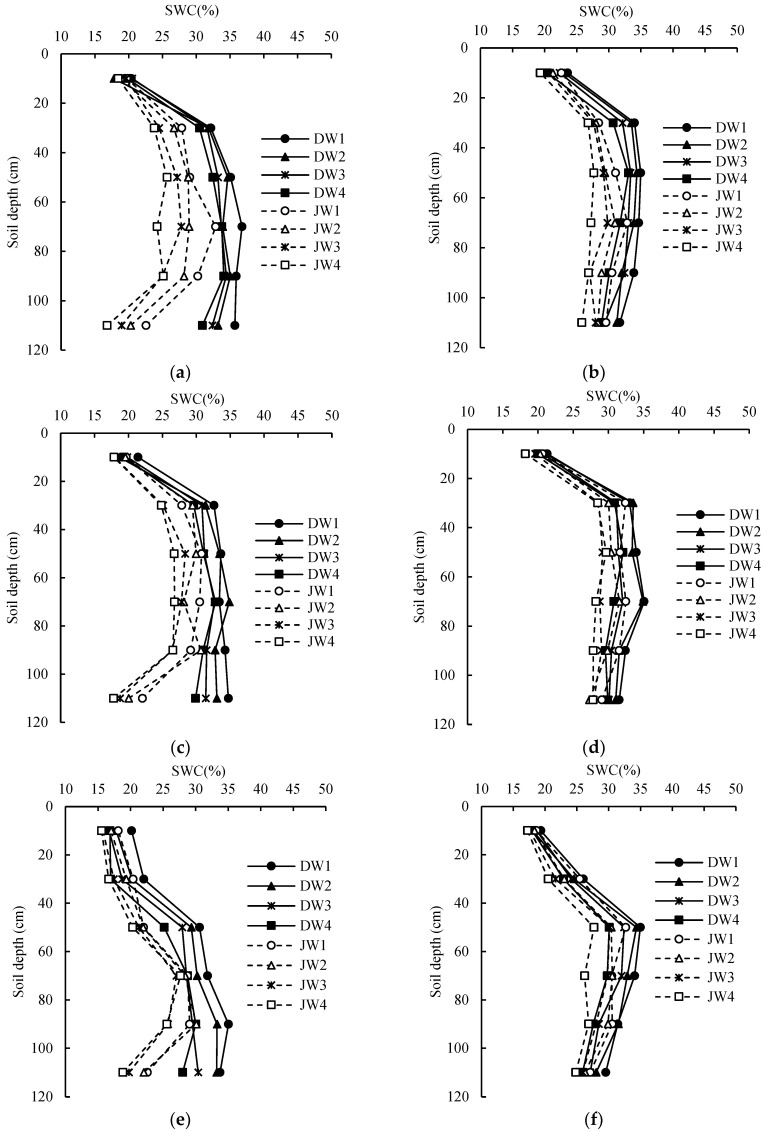

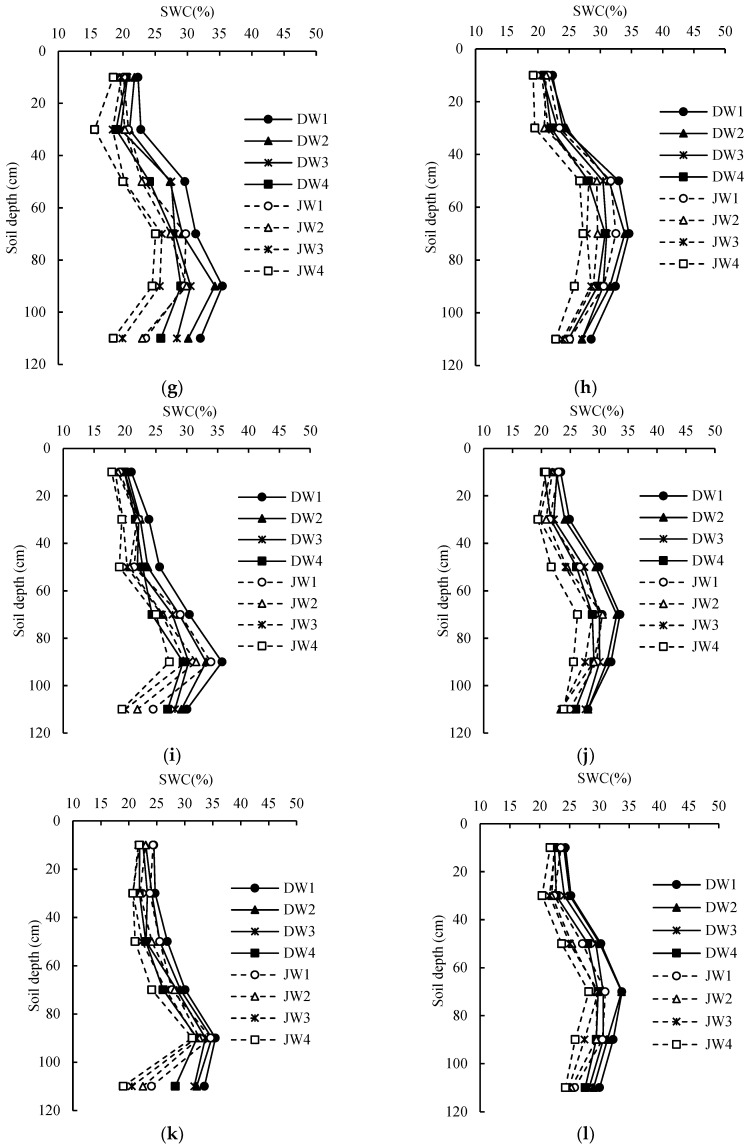
SWC variation at a depth of 120 cm during the germination (**a**,**b**), spring shoot (**c**,**d**), bloom (**e**,**f**), initial fruit (**g**,**h**), full fruit (**i**,**j**), and autumn fruit (**k**,**l**) stages under the different treatments in 2019 (**left**) and 2020 (**right**). DW1: wolfberry monoculture and full irrigation (75–85%θfc), DW2: wolfberry monoculture and mild deficit irrigation (65–75%θfc), DW3: wolfberry monoculture and moderate deficit irrigation (55–65%θfc), DW4: wolfberry monoculture and severe deficit irrigation (45–55%θfc); JW1: wolfberry intercropped with alfalfa and full irrigation (75–85%θfc), JW2: wolfberry intercropped with alfalfa and mild deficit irrigation (65–75%θfc), JW3: wolfberry intercropped with alfalfa and moderate deficit irrigation (55–65%θfc), JW4: wolfberry intercropped with alfalfa and severe deficit irrigation (45–55%θfc).

The temporal and vertical patterns of SWCs remained consistent across different growth stages from 2019 to 2020. Notably, the shallow soil layers exhibited more pronounced fluctuations in the SWCs, with the most significant changes occurring during fruit development. In contrast, the deeper soil layers demonstrated relatively stable moisture levels. Within the surface 0–20 cm soil layer, the SWCs initially ranged from 15% to 20%, primarily because of the combined effects of soil evaporation and water uptake by the crop roots. Early in the season, increased soil surface evaporation led to a decline in the SWCs. However, as the leaves reached full development and ground cover increased, evaporation rates diminished, resulting in soil moisture levels reaching approximately 20–25%. During this period, the depletion of shallow soil moisture was predominantly driven by soil evaporation, and there were no discernible differences in SWCs between the intercropped and monoculture treatments. In the 20–40 cm soil layer, a noteworthy reduction in soil moisture content was observed after the early flowering stage, reaching levels of between 7% and 12%. This phenomenon was attributed to the continuous growth of plant roots, which extended the water-absorbing zone deeper into the soil profile. During this stage, when water resources were limited, intercropped *Lycium chinense* had a more pronounced impact on SWCs due to the regrowth of alfalfa. Consequently, soil moisture levels in the intercropped areas were 12–22% lower than those in the monoculture treatments. After the fruit-setting stage, the SWCs in the 40–60 cm soil layer experienced a slight decrease, with reductions ranging from 2% to 5%. The 0–60 cm soil layer emerged as the primary zone for water uptake by plant roots, supporting fruit development. Conversely, the soil moisture content in the 80–100 cm soil layer remained relatively stable, exhibiting only minor declines during periods of robust plant growth, signifying reduced water extraction by the deeper roots. Intercropping resulted in a more balanced spatial distribution of soil moisture. The introduction of alfalfa between crop rows effectively optimized the utilization of soil moisture in the 0–60 cm soil layer. Notably, the SWCs within this layer in the intercropped plots was consistently lower, ranging from 7% to 19%, compared with the monoculture treatments (Figure 6).

### 2.6. Transpiration

During the budding stage, the plant is in its early growth phase, and water consumption is primarily dominated by soil evaporation with relatively low transpiration rates. In the monoculture treatment, the Transpiration-to-Evapotranspiration ratio (T/ET) stands at only 24.90%, whereas, in the intercropping treatment, it reached 36.87%. As plants enter the vigorous shoot growth phase, leaf development and increased photosynthetic activity led to a rapid rise in transpiration pull. In response, the root system actively absorbs the soil moisture for transpiration. At this stage, the soil moisture content experienced a rapid decline, shifting the water consumption from soil evaporation to plant transpiration. The T/ET values for the monoculture and intercropping treatments were 67.52% and 82.02%, respectively. Transpiration reached its peak and stabilized by the onset of the initial fruiting stage. With fully developed leaves and maximum ground coverage, evaporation rates decrease and plant water uptake is predominantly driven by transpiration. During the initial fruiting and subsequent stages, soil moisture remained consistently low with minimal fluctuations. In the monoculture treatment, the T/ET values were 73.99%, 74.20%, and 71.92% for the initial, peak, and late fruiting stages, respectively. Conversely, the intercropping treatment resulted in T/ET values of 83.58%, 83.22%, and 84.11% for the corresponding stages. Throughout the growth period, the intercropping treatment maintained a T/ET ratio of 76.90%, whereas the monoculture treatment exhibited a lower T/ET ratio of 64.74% (Table 4). The higher T/ET ratio in the intercropping treatment suggests that the spatial arrangement of multiple components was advantageous. It effectively reduced soil evaporation, as discussed earlier, leading to a 32% decrease in ineffective soil evaporation compared with monoculture. Additionally, the intercropping treatment demonstrated a 29% increase in transpiration compared with the monoculture, thereby achieving water-saving effects. Notably, there was no significant difference in the transpiration rates between the monoculture and intercropping treatments in 2019 and 2020.

## 3. Discussion

### 3.1. Crop Coefficients

The variation pattern of crop coefficients during the growth stages is well documented and is characterized by a lower coefficient in the initial growth phase, followed by a linear increase during the rapid growth period. This coefficient reaches its maximum and stabilizes during the mid-growth phase, with a slight decline observed in the late-growth phase [30]. Wolfberry, which are multi-branched shrubs, undergo a relatively short period of rapid growth followed by an extended mid-growth phase [31]. In a study by Yin et al. [32] using the single crop coefficient method, wolfberry exhibited a crop coefficient (K_c_) of from 0.67 to 0.68 during the spring shoot growth period, from 1.14 to 1.16 during the fruit ripening period, and from 0.89 to 0.92 during the leaf-falling period. The dual crop coefficient method dissects K_c_ into two components: the basal crop coefficient (K_cb_) representing crop transpiration and the soil evaporation coefficient (K_e_) representing soil evaporation. In this study, using the dual-crop coefficient method, the baseline crop coefficient (K_cb ini_) for wolfberry in a monoculture was determined to be 0.25. During the mid-growth phase, K_cb mid_ was 0.9, whereas, in the late growth phase, K_cb end_ was 0.6. The soil evaporation coefficient (K_e_) for wolfberry exhibited a range of from 0.43 to 1.02 in the early growth period and from 0.12 to 0.41 in the mid-growth period and beyond. Alfalfa is a perennial forage crop that displays cyclic variations in crop coefficients) [33]. In the research of Liu et al. [34], the first and second crops of alfalfa were found to have K_c ini_ values of 0.4, K_c mid_ values of 0.95 and 1.2, and K_c end_ values of 0.9 and 1.15, respectively. These coefficients followed an “S”-shaped trend, increasing initially and then decreasing within each crop cycle. In the wolfberry berry-alfalfa intercropping system, the crop coefficient dynamics align more closely with alfalfa, particularly during the mid-growth phase when alfalfa cutting exhibits periodic changes. In the initial growth phase, K_cb_ was 0.25, whereas in the mid-growth phase, it reached 1.19. As alfalfa is typically harvested approximately one week after flowering, the crop coefficient of the intercropping system for wolfberry, K_cb_, remained at 0.9. With the second growth of alfalfa, the crop coefficient of the intercropping system exhibited linear growth, reaching 1.15 when alfalfa completely covered the soil surface. After cutting, in the third crop cycle, K_cb_ was 0.9, and it reached 1.13 when the soil surface was entirely covered before harvesting (late growth phase), with a K_cb_ of 1.08.

### 3.2. Soil Evaporation

After rainfall or irrigation, the SWCs initially increased and soil evaporation occurred at its maximum rate. During this phase, soil evaporation is primarily limited by the energy available at the surface. As cumulative evaporation increases, soil moisture decreases, leading to a reduction in soil evaporation rate, a phenomenon known as evaporation decline [35]. In the early stages of crop development, when leaves are not fully formed and ground cover is minimal, soil evaporation rates are high. However, as the leaves reach full development, soil evaporation decreases and only increases again following irrigation or rainfall, exhibiting pulse-like fluctuations. These findings are consistent with those of Li [36], who studied evaporation patterns between corn rows in Tongliao. Li [36] found that, early in the growth period, soil evaporation peaked, especially during the seedling stage, accounting for 85% of total evaporation. As corn plants grow, evaporation rates decrease but increase after irrigation or rainfall events. Similar conclusions were drawn by Duan et al. [37] in their study of wolfberry. Using different models, they estimated soil evaporation during the wolfberry growth period to be between 119.56 mm and 139.72 mm. In contrast, our study found an average soil evaporation of 292.48 mm for monocultured wolfberry farming. This discrepancy may be attributed to the shorter planting duration and wider spacing of wolfberry plants in our study area, resulting in higher soil evaporation owing to the arid climate. Furthermore, we observed that, in wolfberry–alfalfa intercropping systems, the average soil evaporation was 198.68 mm, representing a 32% reduction in soil evaporation compared with monoculture wolfberry farming. These findings align with the research of Huo et al. [38] on apple–canola (*Brassica napus* L.) intercropping systems in the loess hilly region, where intercropping reduced soil evaporation by from 25% to 33%. Intercropping systems featuring tall and short crops can effectively optimize land resource utilization, achieve a more rational spatial layout, reduce soil evaporation, utilize soil moisture between crop rows, and enhance water use efficiency [39]. Moderating irrigation can promote water compensation among intercropped components, encouraging crops to tap deeper into soil moisture [40]. These results were consistent with our findings, indicating that intercropping can mitigate water stress through interactions between different components.

### 3.3. Soil–Crop Water Regulation

The soil–crop system primarily regulates soil moisture distribution through soil evaporation and crop transpiration [41]. Soil evaporation leads to changes in soil moisture, affecting crop water uptake through root systems, which in turn influences crop transpiration. Transpiration is closely linked to crop growth, plant height, and leaf development. These interactions create a complex web of feedback mechanisms within the soil–crop system [42]. Compared to monocultures, intercropping systems involve competition among different crop components in both underground and aboveground spaces. This competition inevitably affects the spatiotemporal dynamics of soil moisture. At the initial stages of growth, there was no significant difference in SWCs between the intercropping and monoculture treatments. During this period, water consumption by crops is primarily driven by soil evaporation, with transpiration accounting for a lower proportion of the total evapotranspiration [43]. At this stage, when leaves were not fully developed and ground cover was minimal, the soil moisture distribution was similar between the intercropping and monoculture treatments. As crops continue to grow, especially in the mid-to-late stages, leaf development becomes complete and transpiration becomes the dominant mode of water consumption, particularly in intercropping treatments. In these cases, both wolfberry and alfalfa absorb significant amounts of soil moisture for transpiration, resulting in soil moisture levels 1–15% higher in monoculture than in intercropping, especially when irrigation amounts are substantial. Our study revealed that, in the early growth stages, the transpiration/evapotranspiration ratio (T/ET) was only 24.90% for monoculture but reached 36.87% for intercropping. In the mid-to-late stages, T/ET ranged from 64.05% to 76.65% for monoculture and from 76.85% to 85.09% for intercropping. On a spatial scale, the main differences in SWCs between intercropping and monoculture treatments occurred in the 0–60 cm soil layer, which is where active root water absorption occurs [44]. There were no significant differences between intercropping and monoculture treatments in the 0–20 cm soil layer. This can be attributed to the fact that the 0–20 cm layer mostly consists of easily evaporable soil [30], in which evaporation is the dominant process and root water uptake plays a secondary role. During the early stages of intercropping, when alfalfa did not provide full ground cover, soil evaporation was high. However, during the mid-to-late stages, alfalfa cover reduced intercropping soil evaporation, whereas crop transpiration increased [45]. During the germination phase, intercropping resulted in SWCs in the 20–40 cm layer, which was 12–22% lower than that in the monoculture. This can be attributed to the fact that, during this phase, alfalfa in intercropping is in its regrowth stage, absorbing a significant amount of soil moisture for its growth, causing lower soil moisture levels in intercropping than in monoculture. In the 40–60 cm layer, the soil moisture content decreased after the fruiting stage because of the transition of wolfberry from vegetative to reproductive growth. During this stage, increased transpiration led to a water potential gradient generated by transpiration pull [46], resulting in a from 2% to 5% decrease in SWCs. Quan et al. [47] found that covering apple orchards with sedges significantly converted soil evaporation into plant transpiration. In line with these findings, our study suggests that wolfberry–alfalfa intercropping, with its more rational spatial distribution, maintains soil moisture levels from 7% to 19% lower than monoculture in the primary root water uptake zone (0–60 cm). This allocation of more water for crop transpiration in intercropping leads to a 29% increase in transpiration compared with monoculture, effectively reducing soil evaporation and enhancing water-use efficiency.

Therefore, through a two-year experiment and an in-depth investigation into the soil–crop water regulation mechanisms within the wolfberry and alfalfa intercropping system, it was found that agroforestry practices could alleviate water stress to a certain extent, achieving water-saving goals. However, this study has yet to provide a clear understanding of root distribution within the intercropping system. Overlapping root systems and competition between components during their growth processes add complexity to the soil moisture distribution. To further elucidate the mechanisms of water regulation, future research should focus on clarifying root distribution within intercropping systems.

## 4. Materials and Methods

### 4.1. Site Description

**Figure 7:**
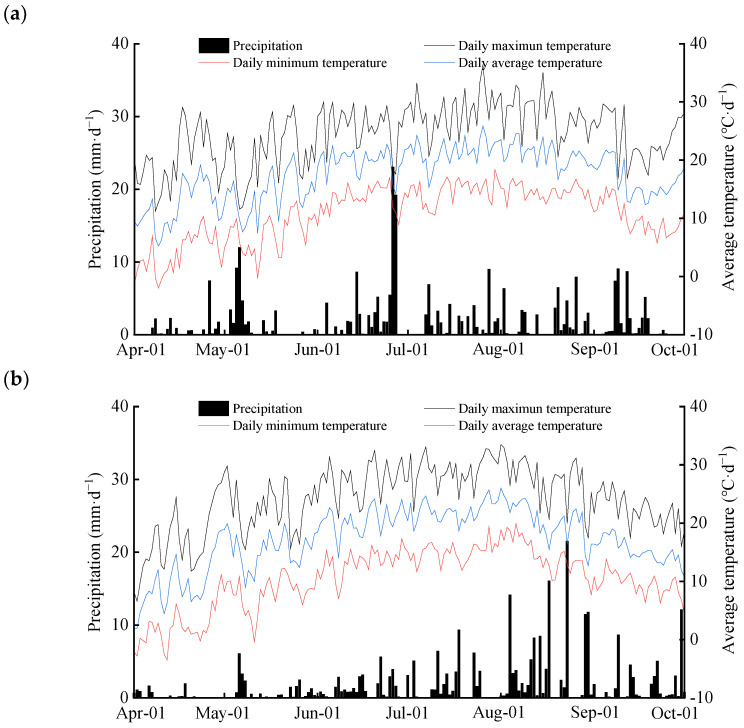
Daily precipitation and average temperature during field experiments in 2019 (**a**) and 2020 (**b**).

This experiment was conducted at the High-Efficiency Water-Saving Irrigation Experimental Station of the Jingtaichuan Electric Power Pumping Management Bureau in Gansu Province, China (37°26′ N, 104°04′ E; average altitude of approximately 1562 m) from 2019 to 2020. The experimental area falls within a temperate arid continental climate zone, which is characterized by low rainfall, with an average annual precipitation of 185 mm. It also experiences high evaporation rates, with an average annual evaporation of 3028 mm. Additionally, the area has significant diurnal temperature variations, with an average annual temperature of 8.3 °C, ranging from a maximum of 35.6 °C to a minimum of −27 °C. The region experiences an annual average of 2652 h of sunlight and an annual average radiation of 6.18 × 10^5^ J cm^−2^. The frost-free period spans 191 days. The soil type in the experimental area was loam with a bulk density of 1.35 g cm^−3^ and a field capacity (θ_fc_) of 24.1% (mass-based water content). The hydraulic characteristics of the soil are listed in Table 5. The soil nutrient content at the experimental site included organic matter at 1.32 g kg^−1^, available nitrogen at 74.51 mg kg^−1^, available phosphorus at 26.31 mg kg^−1^, and a pH value of 8.11. In 2019, the total precipitation and average temperature during the entire growth period of wolfberry were 227 mm and 19.1 °C, respectively. In 2020, during the entire growth period of wolfberry, the total precipitation and average temperature were 101 mm and 19.7 °C (Figure 7).

### 4.2. Experimental Design

**Figure 8:**
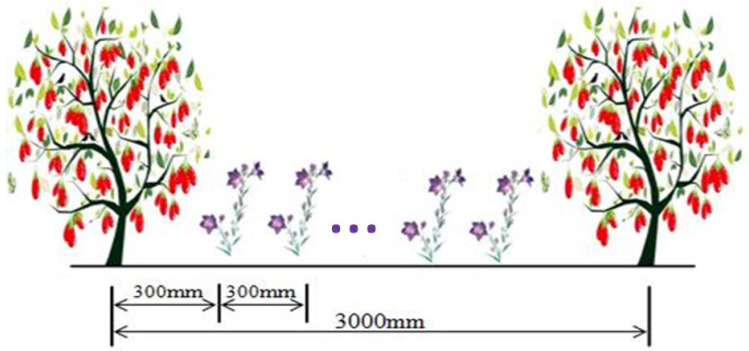
Intercropping pattern.

The experiment used a two-factor randomized block design. Factor one consisted of two planting modes: wolfberry monoculture (D) and wolfberry intercropped with alfalfa (J) (Table 6). Factor two involved water management, in which the irrigation plan targeted soil moisture within a designated range (based on the percentage of field capacity with a moistened layer depth of 60 cm): full irrigation (W1, 75–85%θ_fc_), mild deficit irrigation (W2, 65–75%θ_fc_), moderate deficit irrigation (W3, 55–65%θ_fc_), and severe deficit irrigation (W4, 45–55%θ_fc_). In late April 2017, two-year-old wolfberry seedlings were transplanted with north–south row planting, with a spacing of 150 cm between plants and 300 cm between rows. Simultaneously, alfalfa was strip-sown between the rows of wolfberry (in the north–south direction) at a distance of 30 cm from the tree trunks, with a row spacing of 30 cm (Figure 8). Eight treatment groups were established with three replicates each, totaling 24 plots. Each plot had an area of 45 m^2^ (7.5 m × 6.0 m), with two rows of wolfberry, each containing five plants, making a total of 10 plants per plot. After planting, sufficient water was applied to ensure the survival and growth of both the wolfberry and alfalfa. Water management practices commenced in April 2019, using a drip irrigation system. The field irrigation system included water storage tanks, water pump devices, filtration equipment, main pipes, branch pipes, and micro-tubes (embedded drip irrigation tubes with an inner diameter of 16.0 mm, drip emitter spacing of 0.3 m, and a flow rate of 2.0 L h^−1^). Drip irrigation tapes were placed on both sides of the wolfberry tree trunks at a spacing of 0.3 m. Each experimental plot was equipped with a water meter (precision of 0.0001 m^3^) to control the irrigation volume, and fertilization was performed using a venturi fertilizer applicator.

### 4.3. Measurements and Calculations

#### 4.3.1. Meteorological Data

Meteorological data, including wind speed, atmospheric pressure, relative humidity, precipitation, temperature, solar radiation, and other relevant parameters, were automatically collected at hourly intervals using a compact Davis meteorological station imported from the United States.

#### 4.3.2. Soil Water Content

In each experimental plot, a time-domain reflectometer (TDR) probe extending to a length of 120 cm was positioned 30 cm from the base of the wolfberry plants. This arrangement was replicated three times. Soil water content (SWC), spanning the 0–120 cm soil layer, was systematically measured at 7-day intervals. These measurements were conducted using a PICO-BT TDR instrument, which is a German device manufactured by IMKO. SWCs were calibrated at various growth stages of wolfberry plants using standard soil drilling and oven drying methods. A proactive irrigation regimen was promptly initiated in response to soil moisture levels falling below the predetermined lower threshold as per the specified moisture treatment requirements.

#### 4.3.3. Soil Evaporation

In the plots designated for ample irrigation experiments, automatic inter-row soil evaporation gauges (manufactured by Beijing Time-Domain Technology Co., Ltd., Beijing, China) were placed at a distance of 300 cm from the base of the wolfberry plants. These devices comprised four key components: a solar-powered unit, weighing assembly, outer protective bucket for the soil column, and soil column itself. The soil columns were 25 cm in height and 20 cm in diameter, with data collection intervals set at 1 h intervals. Near the automatic inter-row soil evaporation gauges in the ample irrigation plots, micro-lysimeters (MLSs) were installed in triplicate to cross-verify the data generated by the automatic inter-row soil evaporation gauges. In addition, in other irrigation experiment plots and ample irrigation plots, micro-lysimeters were placed at the same locations for daily soil evaporation data collection. For the micro-lysimeters, Polyvinyl chloride (PVC) pipes were used as the inner and outer containers, each with a length of 10 cm. The outer cylinder has a diameter of 16 cm, whereas the inner cylinder has a diameter of 14 cm. The bottom was fitted with a breathable 500-mesh screen to prevent soil leakage [35]. Weights were recorded daily at 17:00 using an electronic balance with a precision of 0.1 g. To ensure consistency with the in-field soil moisture, the soil was replaced every 5–7 days, and soil replacement was conducted following rainfall or irrigation events.

#### 4.3.4. The SIMDualKc Model

The SIMDualKc (Simulation Dual Crop Coeffcient) model, rooted in the principles of water balance, uses the dual crop coefficient approach to effectively differentiate between two key components within its modeling framework: crop transpiration (*K_cb_·ET*_0_) and soil evaporation (*K_e_·ET*_0_). This model is capable of estimating both soil evaporation and crop transpiration and encompasses various modules, including soil, meteorological, crop, irrigation, and intercropping data [48]. In the process of model application, crop transpiration was determined based on calibrated *K_cb_* values, with the model output providing the value of *K_e_*, signifying daily soil evaporation.

(1) $${ET}_{a}=K_{c}\cdot {ET}_{0}=\left(K_{s}K_{cb}+K_{e}\right){ET}_{0}$$

where *ET_a_* is the actual crop evapotranspiration (mm d^−1^), *K_cb_* is the basal crop coefficient, *K_e_* is the soil evaporation coefficient, K_s_ is the water stress reduction coefficient, and *ET*_0_ is the reference evapotranspiration (mm d^−1^).

The model divides the crop growth period into four stages: initial growth, rapid growth, mid-growth, and late growth. The initial values of crop coefficients for these stages (*K_cb ini_*, *K_cb mid_*, and *K_cb end_*) can be obtained from the FAO-56 recommended table. However, when RH_min_ is not equal to 45% or wind speed (*u*_2_) is not equal to 2 m s^−1^, the following correction formula is applied to adjust *K_cb mid_* and *K_cb end_* when they exceed 0.45, as recommended by [30]:

(2) $$\;\;K_{cb}=K_{cb\;(input)}+\left[0.04\left(u_{2}-2\right)-0.04\left({RH}_{min}-45\right)\right]{\left(\frac{h}{3}\right)}^{0.3}$$

The equation uses *K_cb_* (input) to denote the basic crop coefficient for *K_cb_* mid at a wind speed of *u*_2_ = 2 m s^−1^ and a minimum relative humidity of *RH_min_* = 45%, as referenced in the recommended table. The values of *u*_2_ and *RH_min_* correspond to the observed averages, whereas h represents the average crop height.

Soil evaporation predominantly occurs in regions with moist soil surfaces. It was assumed that soil evaporation beneath the plant canopy was within *K_cb_*·*ET*_0_, denoting soil evaporation as *K_e_*·*ET*_0_. The soil evaporation reached its maximum rate when the soil was adequately moist. However, the available energy for surface evaporation constrains the combined crop coefficient to *K_cb_* + *K_e_* ≤ *K_c max_*. As the soil surface dries, soil evaporation becomes directly proportional to the remaining soil moisture content [49].

(3) $$K_{e}=min(k_{r}\left(k_{c\;max}-K_{cb}\right),f_{ew}K_{cmax})$$

In the equation, *K_e_* represents the soil evaporation coefficient, *K_cb_* is the base crop coefficient, *K_c_* max denotes the maximum *K_c_* value after rainfall or irrigation, *K_r_* is a dimensionless coefficient that depends on the cumulative depth of surface soil evaporation (or water consumption) and affects the reduction in evaporation, and few represents the ratio of bare and wet soil, indicating the percentage of the maximum soil evaporation surface area.

The intercropping crop coefficient *K_c_* was determined based on the surface area covered by the different intercropping crops and the height of the crop plants.

(4) $$K_{c}=\frac{f_{1}h_{1}K_{c1}+f_{2}h_{2}K_{c2}}{f_{1}h_{1}+f_{2}h_{2}}$$

The equation involves *f*_1_ and *f*_2_, which represent the surface areas covered by wolfberry and alfalfa, respectively, whereas *h*_1_ and *h*_2_ represent the heights of the wolfberry and alfalfa plants, respectively.

In this study, SIMDualKc requires that the input data consist of the following:

(1) Crop data: basal crop coefficients (*K_cb_*) of the initial, mid-season, and end-season stages under *RH_min_* = 45% and *u*_2_ = 2 m·s^−1^; fraction of ground cover *f_c_*; plant height h [m]; root depth *Z_r_* [m]; and soil water depletion fraction for non-stress (*p*).
(2) Daily meteorological data: precipitation, *u*_2_ [m·s^−1^], minimum and maximum air temperature, *T_min_* and *T_max_* [°C], *RH_min_* [%] and *P* [mm]; reference evapotranspiration, *ET*_0_ [mm] by calculation.
(3) Soil data: the total available water, *TAW* [mm·m^−1^] refers to the capacity of a soil to retain water available to plants; effective depth of the evaporation layer, *Z_e_* [m]; total evaporable water, *TEW* [mm] and readily evaporable water *REW* [mm].
(4) Irrigation data: fraction of soil wetted by irrigation, irrigation depth, and irrigation date.
(5) Intercropping data included the selected intercropping type, intercropping crop soil coverage ratio, and comprehensive data packages containing information on the intercropping crop, soil, meteorological conditions, and irrigation data.

#### 4.3.5. Statistic Analysis

The SIMDualKc model based on the FAO dual crop coefficient requires input data: meteorological data, soil data, crop data, irrigation data, and intercropping data, and the model parameters are adjusted by giving the initial parameters *K_cb_* and soil water deficit coefficient. The regression coefficient (*b*), coefficient of determination (*R*^2^), root mean square error (*RMSE*), discrete accuracy index of accuracy (*d_IA_*), and model effectiveness coefficient (*EF*) were used to test the simulation effect of the model.

## 5. Conclusions

In conclusion, the SIMDualKc model provided accurate simulations of soil moisture and evaporation for both monoculture and intercropping systems of wolfberry and alfalfa. For monoculture wolfberry, the crop coefficients (*K_cb_*) followed a pattern with values of 0.25 during the early growth stage (*K_cb ini_*), 0.9 during the mid-growth stage (*K_cb mid_*), and 0.6 in the late growth stage (*K_cb end_*). During the rapid growth period, the soil evaporation coefficient (*K_e_*) ranged from 0.85 in the early growth stage to 0.27. In the intercropping system, influenced by alfalfa growth characteristics, the crop coefficients exhibited a cyclic pattern. During the early growth stage, *K_cb ini_* was 0.25, which varied during harvests, reaching an average of 1.19 before each harvest, and decreased to 0.9. This pattern continued through the three cycles, resulting in a balanced distribution of soil moisture. The intercropping system effectively reduced soil evaporation by 34%, with soil evaporation decreasing from 292.48 mm in the monoculture to 198.68 mm in the intercropping system. This spatial planting strategy enhanced water use efficiency, with transpiration rates in intercropping being 29% higher than that in monoculture, demonstrating water-saving benefits.

## Figures and Tables

**Table 1:** Initial and calibrated parameters used in SIMDualKc model.

Parameters			Initial	Calibrated
Wolfberry	Crop	K_cb ini_	0.15	0.25
		K_cb mid_	1.1	0.9
		K_cb end_	0.7	0.6
		P_ini_	0.45	0.45
		P_mid_	0.45	0.5
		P_end_	0.45	0.45
	Soil evaporation	TEW	28	28
		REW	12	12
		Z_e_	0.15	0.15
Alfalfa	Crop	K_cb ini_^1^	0.3	0.4
		K_cb mid_^1^	1.15	1.25
		K_cb end_^1^	1.1	1.2
		p_ini_	0.55	0.55
		p_mid_	0.55	0.5
		p_end_	0.55	0.55
	Soil evaporation	TEW	28	28
		REW	12	12
		Z_e_	0.15	0.15

These K_cb_ coefficients for hay crops represent immediately following cutting, at full cover, and immediately before cutting. The growing season was described as a series of individual cutting periods. K_cb ini_—basal crop coefficient for the initial crop development stage, K_cb mid_—basal crop coefficient for the mid-stage, K_cb end_—basal crop coefficient for the late-season stage, p—depletion fraction (ini—initial crop; mid—mid-stage; end—late-season stage), TEW—total evaporable water (mm), REW—readily evaporable water (mm), and Z_e_—thickness of the evaporation layer (m).

**Table 2:** Parameters of the calibrated and verified soil moisture content data.

Year	Treatment	b	R^2^	RMSE (m^3^·m^−3^)	d_IA_	EF
2019 (Calibration)	intercropping	0.99	0.89	0.0267	0.92	0.70
	monoculture	0.99	0.88	0.0163	0.93	0.70
2020 (Validation)	intercropping	1.00	0.86	0.0193	0.92	
	monoculture	0.98	0.87	0.0151	0.93	

**Table 3:** Parameters of the calibrated and verified soil evaporation data.

Year	Treatment	b	R^2^	RMSE (mm)	d_IA_	EF
2019 (Calibration)	monoculture	0.89	0.93	0.51	0.96	0.84
	intercropping	0.93	0.91	0.54	0.95	0.82
2020 (Validation)	monoculture	0.89	0.87	0.55	0.92	
	intercropping	0.83	0.93	0.50	0.94	

**Table 4:** Transpiration (T) and transpiration ratio (T/ET) for each growth stage in the monoculture and intercropping treatments.

Treatment	Growth Stage	2019		2020	
		T (mm)	T/ET (%)	T (mm)	T/ET (%)
monoculture	germination stage	32.634	23.85%	32.75	25.94%
	spring shoot stage	55.119	66.86%	50.96	68.18%
	bloom stage	97.839	76.65%	105.75	71.66%
	initial fruit stage	76.221	75.97%	89.88	72.00%
	full fruit stage	154.12	76.39%	148.95	72.01%
	autumn fruit stage	75.132	73.97%	88.33	69.89%
	whole growth stage	491.07	65.43%	516.63	64.05%
intercropping	germination stage	52.56	37.37%	42.66	36.36%
	spring shoot stage	83.13	83.32%	74.39	80.71%
	bloom stage	113.32	81.98%	127.4	82.11%
	initial fruit stage	92.58	85.09%	100.66	82.06%
	full fruit stage	175.98	83.70%	171.84	82.73%
	autumn fruit stage	111.48	83.56%	118.28	84.66%
	whole growth stage	649.05	76.94%	655.23	76.85%

**Table 5:** Main soil hydraulic properties.

Layer	Depth (cm)	Field Capacity (cm^3^ cm^−3^)	Wilting Point (cm^3^ cm^−3^)
1	0–20	0.27	0.10
2	20–40	0.23	0.10
3	40–60	0.34	0.12
4	60–80	0.36	0.14
5	80–100	0.38	0.14
6	100–120	0.37	0.14

**Table 6:** Experimental design.

Treatment	Monoculture				Intercropping			
	FI	MDI	MDI	SDI	FI	MDI	MDI	SDI
Upper and lower bound for irrigation	75–85%θ_fc_	65–75%θ_fc_	55–65%θ_fc_	45–55%θ_fc_	75–85%θ_fc_	65–75%θ_fc_	55–65%θ_fc_	45–55%θ_fc_
Number	DW1	DW2	DW3	DW4	JW1	JW2	JW3	JW4

FI—Full irrigation, MDI—Mild Deficit irrigation, MDI—Moderate Deficit irrigation, SDI—Severe deficit irrigation.

## Data Availability

All data are incorporated into the article.
